# Mapping abnormal subcortical brain morphometry in an elderly HIV + cohort

**DOI:** 10.1016/j.nicl.2015.10.006

**Published:** 2015-10-08

**Authors:** Benjamin S.C. Wade, Victor G. Valcour, Lauren Wendelken-Riegelhaupt, Pardis Esmaeili-Firidouni, Shantanu H. Joshi, Boris A. Gutman, Paul M. Thompson

**Affiliations:** a Imaging Genetics Center, University of Southern California, Marina del Rey, CA, USA; b Memory and Aging Center, Dept. of Neurology, University of California, San Francisco, CA, USA; c Ahmanson-Lovelace Brain Mapping Center, Department of Neurology, UCLA, Los Angeles, CA, USA

**Keywords:** RD, radial distance, JD, Jacobian determinant, CD4, cluster of differentiation, HIV, Subcortical, Shape analysis, MRI, Random forest, Classification

## Abstract

Over 50% of HIV + individuals exhibit neurocognitive impairment and subcortical atrophy, but the profile of brain abnormalities associated with HIV is still poorly understood. Using surface-based shape analyses, we mapped the 3D profile of subcortical morphometry in 63 elderly HIV + participants and 31 uninfected controls. The thalamus, caudate, putamen, pallidum, hippocampus, amygdala, brainstem, accumbens, callosum and ventricles were segmented from high-resolution MRIs. To investigate shape-based morphometry, we analyzed the Jacobian determinant (JD) and radial distances (RD) defined on each region's surfaces. We also investigated effects of nadir CD4 + T-cell counts, viral load, time since diagnosis (TSD) and cognition on subcortical morphology. Lastly, we explored whether HIV + participants were distinguishable from unaffected controls in a machine learning context. All shape and volume features were included in a random forest (RF) model. The model was validated with 2-fold cross-validation. Volumes of HIV + participants' bilateral thalamus, left pallidum, left putamen and callosum were significantly reduced while ventricular spaces were enlarged. Significant shape variation was associated with HIV status, TSD and the Wechsler adult intelligence scale. HIV + people had diffuse atrophy, particularly in the caudate, putamen, hippocampus and thalamus. Unexpectedly, extended TSD was associated with increased thickness of the anterior right pallidum. In the classification of HIV + participants vs. controls, our RF model attained an area under the curve of 72%.

## Highlights

• We model subcortical morphometry of elderly HIV + participants.
• We explore classifying HIV status based on shape and volume of brain regions.
• Morphometry of brain regions was associated with infection status and duration.
• HIV status was classifiable with 72% accuracy in morphometry-based classifiers.

## 1 Introduction

Combined antiretroviral therapy (cART) has vastly improved the quality of life for people infected with human immunodeficiency virus (HIV), allowing many to live to an advanced age. The over 55 demographic is the most rapidly growing age group of HIV + individuals (Hall et al., 2008). Yet, following the increased life expectancy, numerous reports studying chronic infection indicate that HIV positivity independently contributes to and synergistically exacerbates age-related neurodegeneration (Canizares et al., 2014, Cohen et al., 2015) which in many cases results in dramatic cognitive declines and decreased quality of life, with upwards of 50% proceeding to develop a degree of HIV-associated neurocognitive disorders (HAND) (Cysique et al., 2004, Simioni et al., 2010). Several potential mechanisms for neuronal atrophy have been reported including chronic inflammation, interrupted metabolic processes, immunological senescence, heightened risk for cardiovascular disorders and even side effects of cART. For a more detailed review of these factors we refer the reader to Canizares et al. (2014).

Numerous studies support the view that central nervous system infection parallels the aging process. The Multicenter AIDS Cohort Study (MACS), implementing a longitudinal study with a 5-year follow-up, identified an interaction between HIV status, age and time whereby middle-aged and elderly infected participants exhibited a significant decline in executive function over time relative to uninfected controls who showed no reduction in performance with respect to time (Sacktor et al., 2010). In contrast, (Cysique et al., 2011) observed that age and HIV status independently contributed to cognitive decline but were unable replicate an interaction between the two.

Literature from structural MRI widely reports age disproportionate degrees of atrophy among HIV + cohorts (Becker et al., 2012, Thompson et al., 2005, Towgood et al., 2012) have reported both HIV-related reductions in frontal gray matter. HIV and age associated reductions in subcortical structures are also widely reported (Ances et al., 2012). Diffusion tensor imaging has revealed interactive effects of HIV status and age in the reduction of fractional anisotropy and increase of mean diffusivity within subcortical white matter and frontal regions (Chen et al., 2009). Magnetic resonance spectroscopy reports also suggest that CNS HIV infection mimics the effects of aging. (Chang et al., 2004, Harezlak et al., 2011) both reported higher choline compounds (CHO) : total creatine (CR) and myoinositol (MI) : CR ratios in the basal ganglia associated with aging and HIV status.

Given the complications presented by the synergistic effects of aging and HIV, it is important to further understand the morphological differences that occur between normal aging and aging influenced by HIV infection. While several studies have investigated volumetric differences in elderly HIV + cohorts, a thorough description of shape differences in elderly HIV + subjects remains to be explored. Local descriptions of shape variation may offer an additional characterization of HIV-related neurodegeneration by describing localized regions of tissue atrophy and expansion. This level of description could be leveraged as an additional biomarker for the detection of aberrant degeneration, allowing clinicians to offer more targeted interventions when needed.

We report on both the subcortical volumetric and 3D surface-based shape abnormalities in a cohort of 63 elderly HIV + subjects scanned with high-resolution structural magnetic resonance imaging (MRI) as part of the UCSF (University of California, San Francisco) HIV Over 60 Cohort study. To study subcortical shape, we analyzed: (1) the log of the Jacobian determinant (JD) indexed over structures' surface coordinates and (2) radial distances (RD) of structure surfaces from a medial curve. JD maps indicate localized atrophy or dilation of a surface area. Complementary to JD, RD indicates the local “thickness” of the structure. We tested associations of morphological descriptors with neuropsychological measures: the Wechsler Adult Intelligence Scale (WAIS) and Wide Range Achievement Test (WRAT) scale. We also tested for associations with several common HIV clinical indices: nadir CD4 + T-cell count (nCD4), time since diagnosis (TSD; in years), and HIV RNA level in plasma. We hypothesized that subcortical shape analysis would reveal regions of significant atrophy in HIV + people relative to matched controls and in relation to clinical markers of HIV.

We additionally explored the use of shape and volume features in a machine learning framework to classify participants as HIV + or HIV − using a random forest (RF) classifier; a class of supervised machine learning algorithms that has gained popularity for its accuracy, ease of use, and computational efficiency. Here, the application of machine learning based on brain morphometry is not intended as a potential means of clinical HIV diagnosis; rather the ability to robustly distinguish HIV status based on brain-derived measures would validate that observed differences are highly associated with infection status and potentially relevant to the prediction of further HIV-related neurological atrophy. To our knowledge this is the first study to explore classification of HIV status based solely on brain morphometry.

## 2 Methods

### 2.1 Subjects

A sample of 63 elderly HIV + subjects (2 female; age = 64.68 ± 4.57) and 31 uninfected elderly controls (4 female; age = 65.35 ± 2.21) were recruited as part of a San Francisco Bay Area study of elderly people with HIV. HIV + participants had an average nCD4 count of 204.96 ± 154.85 cells/mm^3^, an average TSD of 20.39 years ± 6.31 years. 24 HIV + participants had detectable levels of viral RNA (above 50 copies/mm^3^). Among those with detectable HIV RNA, the average viral load was 16,380.58 ± 76,418.68 copies/mm^3^. All subjects gave informed consent to take part in the study. Table 1 outlines the demographic and clinical characteristics of the participants.

### 2.2 Image acquisition

Each subject underwent a whole-brain high-resolution magnetic resonance imaging (MRI) anatomical brain scan on a Siemens 3 Tesla TIM Trio scanner with a 12-channel head coil. T1-weighted MP-RAGE sequences (240 × 256 matrix; FOV = 256 mm; 160 slices; voxel size = 1.0 × 1.0 × 1.0 mm^3^; TI = 900 ms; TR = 2300 ms; TE = 2.98 ms; flip angle = 9°).

### 2.3 Morphological descriptors

Previously validated FreeSurfer (Fischl et al., 2002) workflows, including non-brain tissue removal, intensity normalization and automated volumetric parcellation based on probabilistic information from manually labeled training sets, were used to segment the bilateral thalamus, putamen, pallidum, amygdala, accumbens, caudate and hippocampus from the raw MRIs. All segmentations were visually inspected to ensure their quality.

The parameterization of each surface was obtained using the “medial demons” method detailed in (Gutman et al., 2012, Gutman et al., 2015). Briefly, each surface was conformally mapped to the spherical domain. The spherical maps were rigidly rotated to a probabilistic atlas. Next, Spherical Demons (SD) (Gutman et al., 2013) was used to non-linearly register the spherical maps on the basis of curvature. Two surface-based functions were defined to do this; first, the global orientation function, defining the direction of the surface and, secondly, the local thickness of the surface with respect to a skeletonized medial core. Finally, SD was implemented again using both the newly defined medial core in conjunction with surface-based curvature to match each surface to the atlas.

From this process, two shape features are defined at each vertex: 1) radial distance (RD), a proxy for thickness and 2) the log of the Jacobian determinant (JD) which indicates surface dilation or atrophy. Among all 14 brain region surfaces, there were a total of 27,120 vertices.

### 2.4 Statistical methods

Multiple linear regression was used to model influences of HIV status, nCD4 count, viral load, TSD, HAND status and drug abuse history on the morphometry of each surfaces. The general linear model assumed the following form,

(1) $$Y=\beta_{0}+\beta_{1}\cdot \mathrm{Main}\;\mathrm{Effect}+\beta_{2}\;\cdot \mathrm{Age}+\beta_{3}\;\cdot \mathrm{Sex}+\;\beta_{4}\cdot \;ICV+\epsilon$$

where *Y* is global volume, for one of the regions, or the locally computed JD or RD; Main Effect is one of HIV status, nCD4 count, viral load or TSD, HAND or drug abuse history. This model was fitted at each of the surface vertices when the outcome of interest was the shape measure, JD or RD. HIV status and viral load were each modeled dichotomously; HIV status was coded as positive or negative and viral load as detectable (above 50 viral RNA copies/mm^3^) or undetectable (i.e., binary). nCD4 and TSD were modeled continuously. HAND status and drug abuse history were modeled as positive or negative. HAND encompasses a range of impairments including asymptomatic neurocognitive impairment (ANI), mild neurocognitive disorder (MND) and HIV-associated dementia (HAD); a subject having any of these was considered HAND positive in the regression model. Similarly, due to the small number of subjects having a history of drug abuse we simply model any of, marijuana, cocaine, crack or methamphetamine as having a history of abuse.

Associations of morphometry and cognitive measures were modeled using the following general linear model,

(2) $$\begin{array}{l} Y=\beta_{0}+\beta_{1}\cdot \mathrm{Main}\;\mathrm{Effect}+\beta_{2}\;\cdot HIV\;\mathrm{Status}+\beta_{3}\cdot \mathrm{Age}+\beta_{4}\;\cdot \;\mathrm{Sex}\\ +\beta_{5}\cdot ICV+\;\beta_{5}\cdot \left(\mathrm{Main}\;\mathrm{Effect}\cdot HIV\;\mathrm{Status}\right)+\epsilon \end{array}$$

where Main Effect is one of WAIS or WRAT score modeled continuously and HIV Status is a dichotomous term.

We controlled for multiple comparisons using the standard false discovery rate (FDR) method with a false-positive rate of 5% (*q* = 0.05) (Benjamini and Hochberg, 1995). FDR was performed separately for volumetric and shape-based tests. For the family of volumetric tests, FDR was applied to the set of all subcortical structures. For shape analyses we applied an FDR correction within the family of all tests performed on a single surface; correcting for separate tests within each surface.

### 2.5 Random forest classification

In addition to mapping differences in subcortical morphometry, we wanted to investigate the efficacy of our morphometric descriptors as input features in a machine learning, classification context. Here, we describe the RF framework used in this study.

Developed and detailed by (Breiman, 2001), RFs are supervised classifiers composed of an ensemble of classification and regression trees (CART) and use the majority vote of its terminal nodes to predict the class of a given observation. RF CARTs are constructed from a bootstrapped sample of approximately 2/3 of the original observations. At each node of the CART, a random subset of $\sqrt{M}$ features is assessed. Here the Gini impurity index is calculated for each feature at the given node, v. Gini(v) is given by

(3) $$\mathrm{Gini}\left(v\right)=\sum_{C=1}^{C}{\hat{p}}_{c}^{v}\left(1-{\hat{p}}_{c}^{v}\right)$$

where ${\hat{p}}_{c}^{v}$ is the proportion of observations belonging to class C at node v. The objective of the RF algorithm is to split each CART node by the feature *X*_*i*_ which maximizes the class purity of the resultant child nodes, *v*^*r*^ and *v*^*l*^. This is done by choosing the maximum Gain(X_i_, v) given by,

(4) $$\mathrm{Gain}\;\left(X_{i},v\right)=\;\mathrm{Gini}\left(X_{i},v\right)-\;\omega_{l}\mathrm{Gini}\left(X_{i},v^{l}\right)-\omega_{r}\mathrm{Gini}\left(X_{i},v^{r}\right)\text{,}$$

where *ω*_*l*_ and *ω*_*r*_ are the proportions of observations in node v assigned to child nodes *v*^*r*^ and *v*^*l*^, respectively. The importance I, of feature *X*_*i*_ is given by the summation of the decreases in the Gini index at each node where the CART was partitioned by *X*_*i*_ (Gray et al., 2013). That is,

(5) $$I_{X_{i}}=\;\frac{1}{\mathrm{total}\;\mathrm{tree}\;\mathrm{number}}\;\sum_{v\in S_{X_{i}}}\mathrm{Gain}\left(X_{i},v\right)\text{,}$$

where $S_{X_{i}}$ indicates the set of all nodes split by *X*_*i*_. Each CART was grown to its full, unpruned extent.

Our RF model was implemented in R (R. Core Team, 2014) and used the RRF package (Deng, 2013). The RF was composed of 5000 trees. We trained the model on half of the participants, stratified by HIV status, using the remaining half for cross validation. The training set consisted of 28 HIV + and 15 HIV − participants while the test set included 27 HIV + and 15 HIV − participants. The RF model was constructed using a combination of all morphological features; all volumetric, RD and JD values were entered as predictors of HIV status.

The significance of the RF was assessed using a permutation test. This was done by first computing the observed area under (AUC) the receiver operating characteristic curve (ROC) from the prediction of the test set. This obser+ved AUC was compared to a null distribution of 1000 AUC values resulting from the classification of HIV status based on randomly shuffling the labels associated with the observed prediction. The proportion of AUCs in the null distribution that were larger than the observed AUC is the p-value associated with the null hypothesis that the observed AUC is less than or equal to 50%, i.e. classification is no better than chance.

As a follow-up analysis we constructed RF classifiers on feature sets composed uniquely of either RD, JD or volumetric measures. We corrected for the set of all classifier p-values using FDR correction for multiple comparisons.

## 3 Results

We found several associations between subcortical morphometry and HIV status and clinical parameters. In the following sections we outline the observed morphometry associated with HIV status, nadir CD4 count, detectability of viral load and TSD.

### 3.1 HIV status

**Fig. 1:**
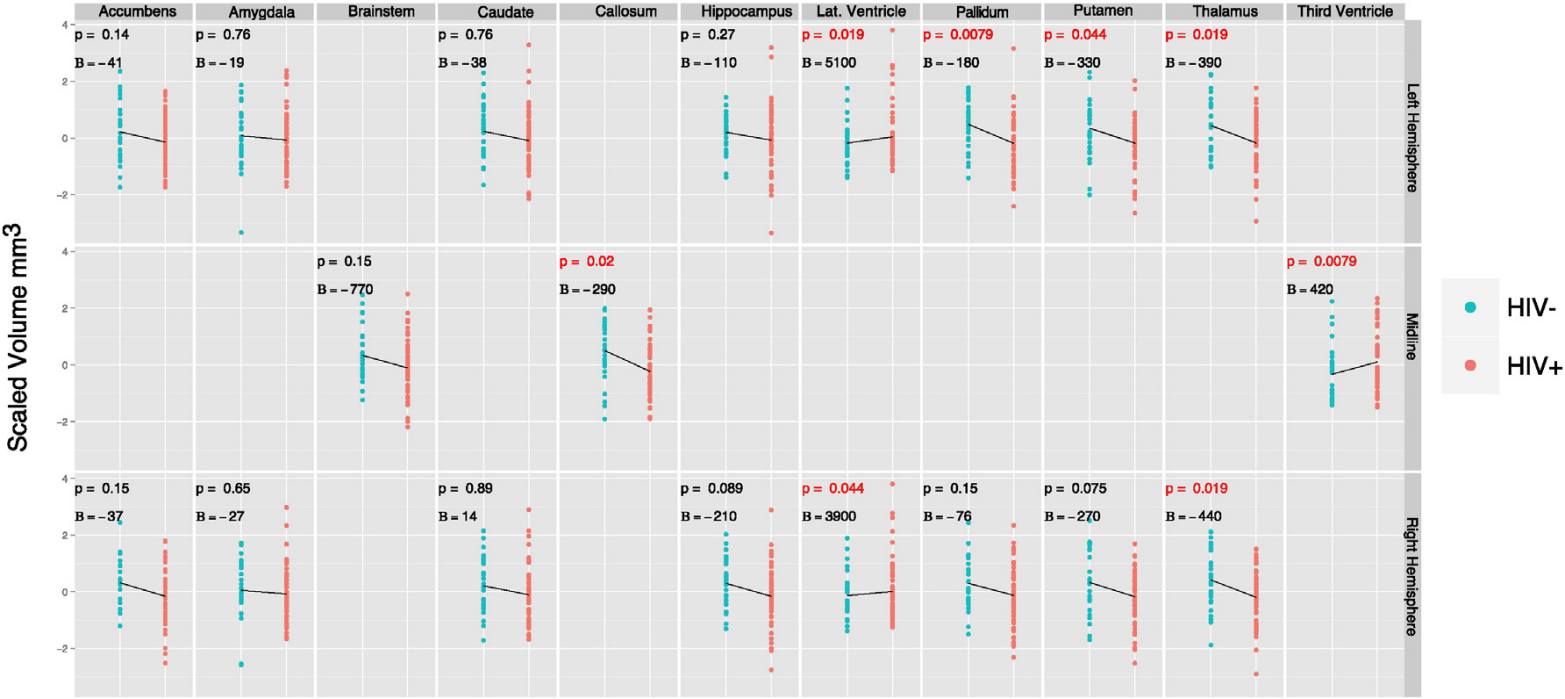
Scatterplot of volumetric differences between HIV + and HIV − participants. The plots are divided into rows showing structures in the left hemisphere (top), midline (middle) and right hemisphere (bottom). All plots have been centered and scaled. The HIV status coefficient (in mm^3^) resulting from Eq. (1) is given for each plot. Volumetric differences that survive FDR correction are highlighted in red.

Volumetrically, the callosum (*β*_*dx*_ = − 290, − 10.7%, t = − 2.81, p < 0.05), left pallidum (*β*_*dx*_ = − 180, − 7.6%, t = − 3.47, p < 0.01), left putamen (*β*_*dx*_ = − 330, − 5.7%, t = − 2.40, p < 0.05), left thalamus (*β*_*dx*_ = − 390, − 5.9%, t = − 2.91, p < 0.05) and right thalamus (*β*_*dx*_ = − 440, − 6.3%, t = − 3.00, p < 0.05) were all significantly smaller in HIV + participants. Ventricular spaces were, on average, enlarged in HIV + subjects. Specifically, the left lateral (*β*_*dx*_ = 5100, 12.7%, t = 2.88, p < 0.05), right lateral (*β*_*dx*_ = 3900, 8%, t = 2.41, p < 0.05) and third (*β*_*dx*_ = 420, 15.5%, t = 3.68, p < 0.01) ventricular spaces were all significantly larger in HIV + participants. Fig. 1 illustrates volumetric differences between HIV + and HIV − participants. All units for volumetric measures are in mm^3^.

**Fig. 2:**
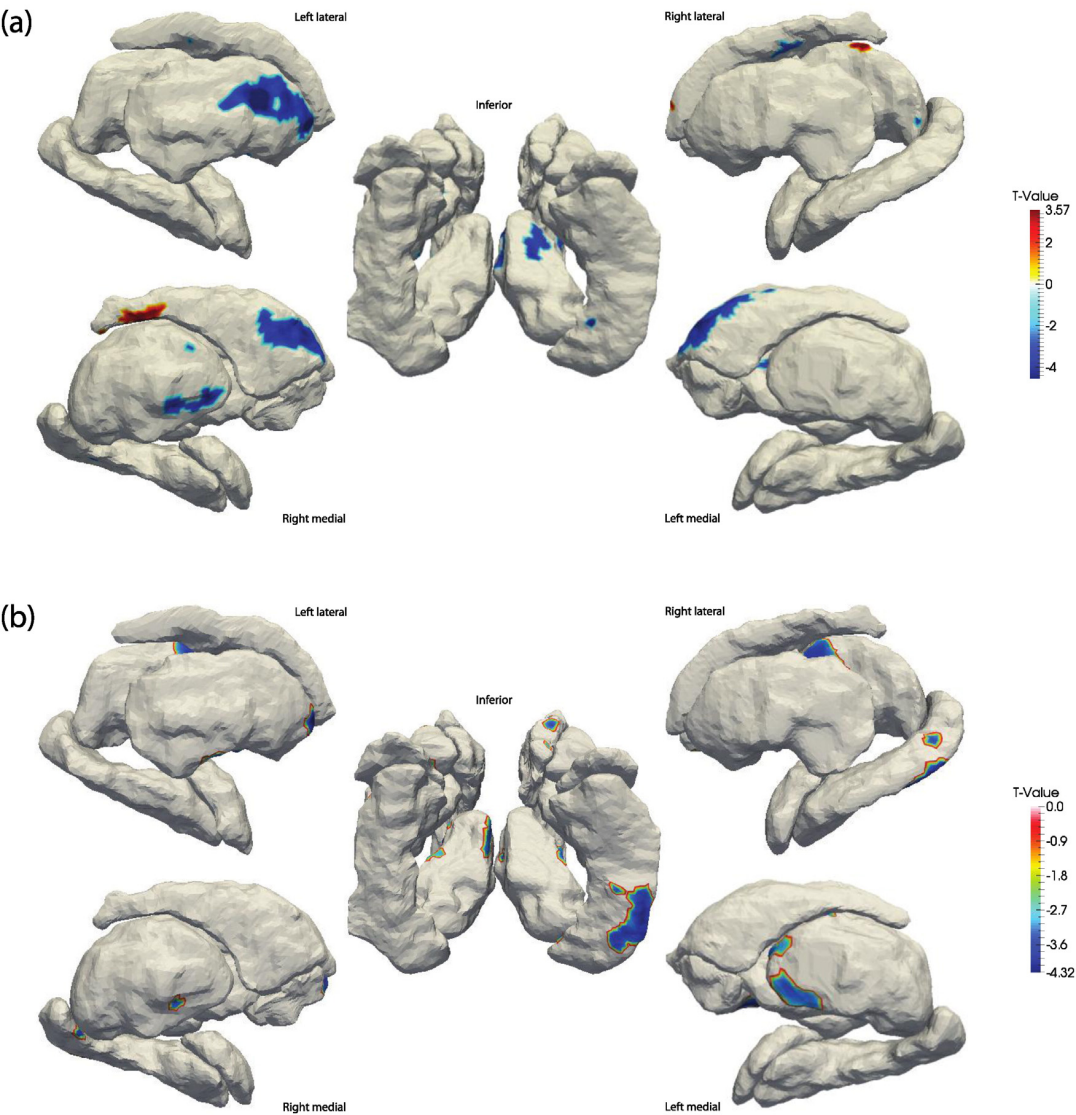
FDR-thresholded t-value maps of the HIV + vs. HIV − contrast. (a) maps the significant differences in RD while (b) maps significant differences in JD. Images are shown in radiological orientation (i.e. left-right flipped), orientations are provided beside each set of surfaces.

Shape mapping revealed widespread regions of atrophy in the HIV + cohort. Specifically, RD maps, illustrated in Fig. 2(a), identified significant atrophy of the bilateral medial aspect of the caudate head; regions of atrophy and expansion were observed in the posterior tail regions as well. Several regions of the bilateral pallidum, left putamen and right inferior thalamus and inferior hippocampus were also significantly atrophied in HIV + participants. JD mapping, depicted in Fig. 2(b), corroborated the observed widespread atrophy in a set of regions only partially overlapping with those found in the RD maps. The JD maps highlight atrophy laterally and medially in the bilateral thalamus, anteriorly in the left putamen and right accumbens and in the inferior–posterior right hippocampus. Morphometry differences outside of these regions were below the set statistical threshold of significance.

### 3.2 Nadir CD4 + counts

There were no detectable volumetric or shape-based associations between nCD4 counts and morphometry.

### 3.3 Viral load

No significant shape or volume differences were found between HIV + participants with and without detectable viral RNA levels.

### 3.4 Time since diagnosis

**Fig. 3:**
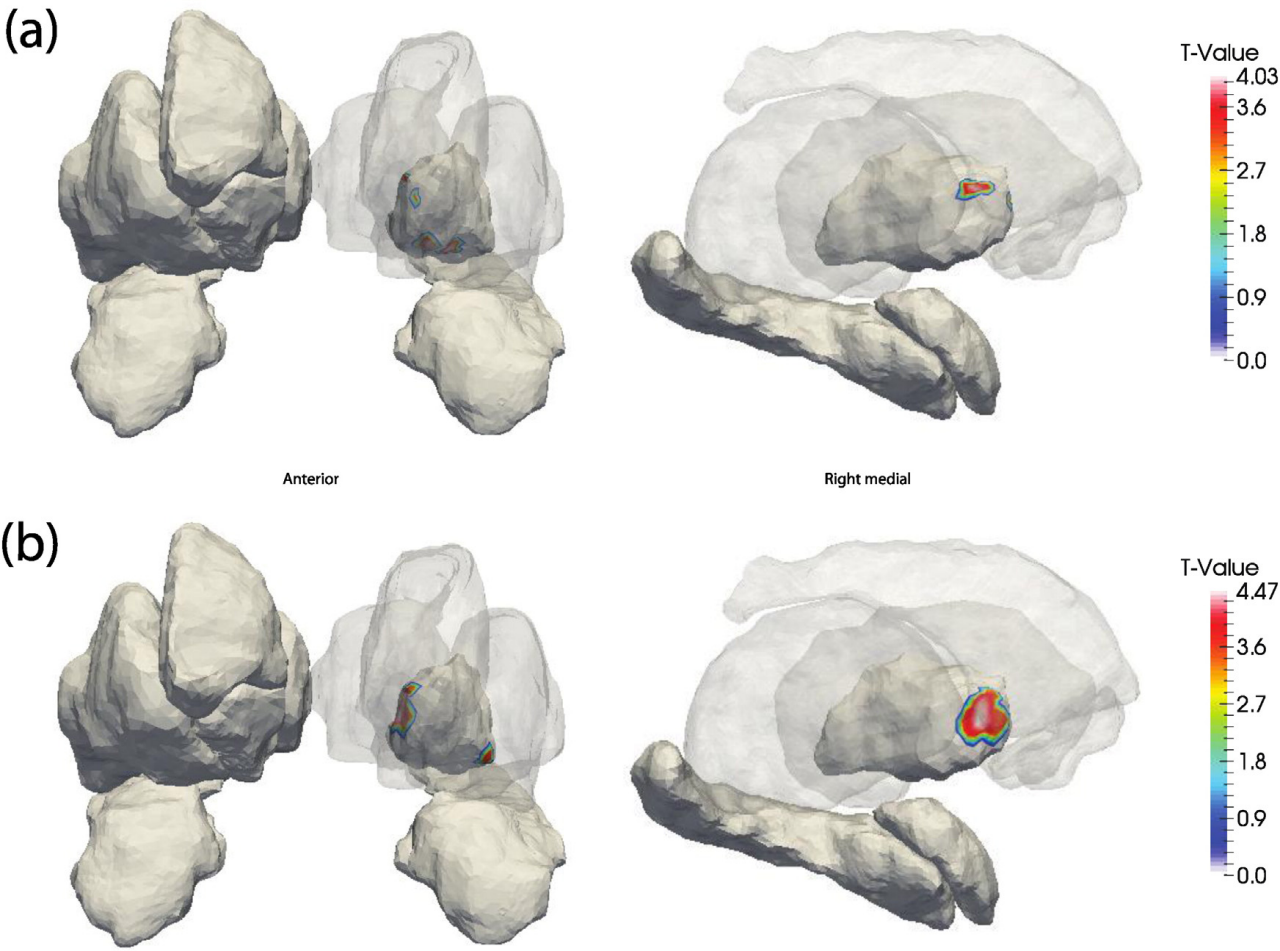
FDR-thresholded t-value maps of the association between TSD and (a) RD and (b) JD. Images are in radiological orientation (i.e. left–right flipped). The first column shows an anterior view while the second is the medial view of the right hemisphere structures.

The volume of the right pallidum (*β*_*duration*_ = 6.3, t = 2.43, p > 0.05) was positively associated with TSD prior to correction for multiple comparisons but failed to survive FDR. Corroborating this, both RD and JD maps of the right pallidum indicate significant local expansion in its anterior aspect in relation to increased TSD; RD and JD results survived FDR (see Fig. 3).

### 3.5 Drug abuse history

No significant associations were found between brain morphometry and drug abuse history.

### 3.6 HIV-associated neurocognitive disorder

HAND revealed no observable associations with brain morphometry.

### 3.7 Cognitive measures

**Fig. 4:**
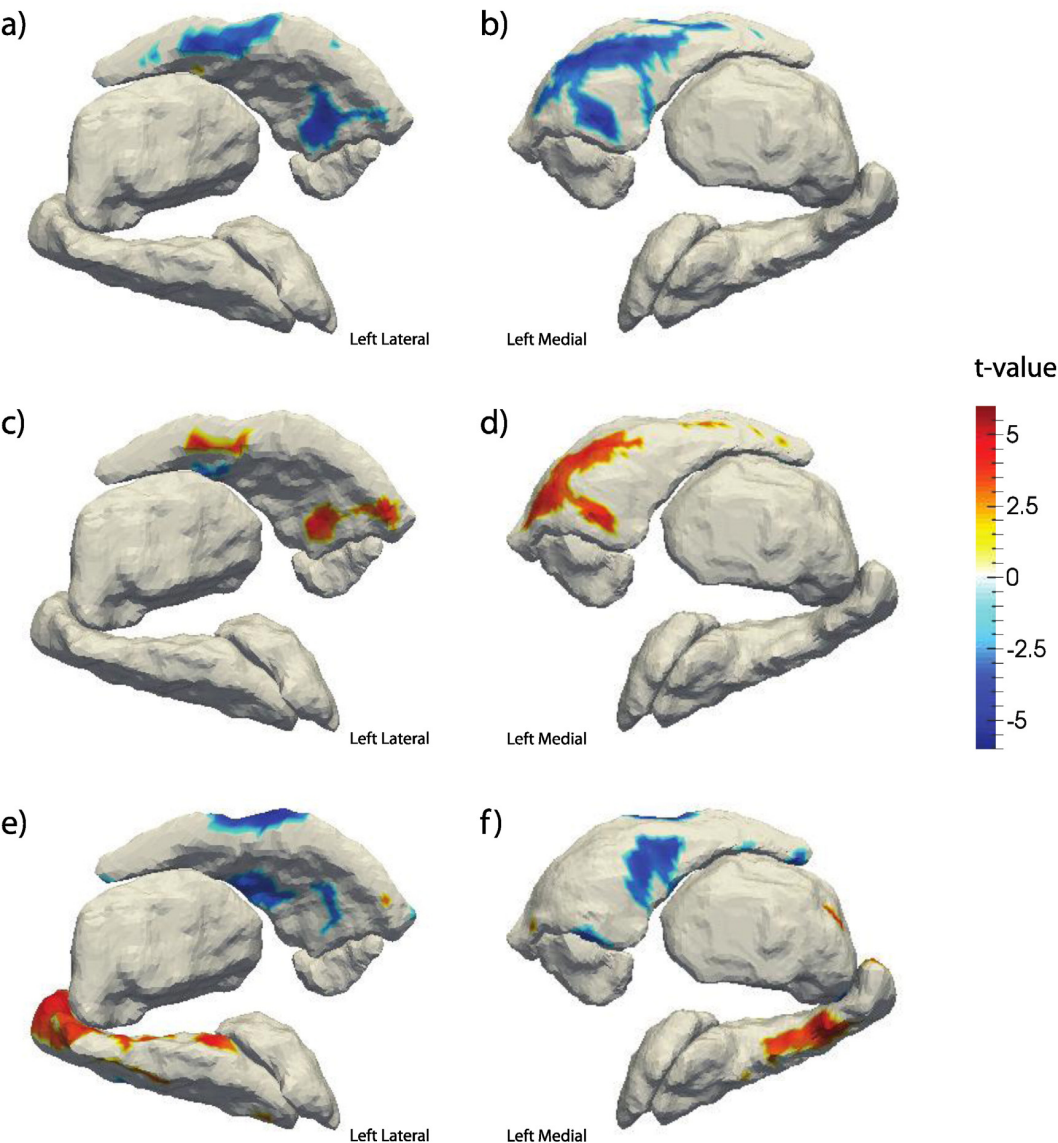
FDR-thresholded t-value maps of (a–b) association of thickness and the interaction of WAIS score and HIV Status, (c–d) the main effect of WAIS on thickness and (e–f) the association of WAIS and thickness within the HIV + cohort only.

Both WAIS and WRAT scores were significantly larger in the HIV − cohort, t(54) = − 3.97, and t(70) = − 3.43, respectively, both p < 0.001. The interaction of HIV status and WAIS or WRAT score was not significantly associated with volume. However, the thickness of the left caudate was widely associated with the interaction of HIV status and WAIS score. Fig. 4 maps both the main effect of WAIS score (c–d) and the interaction of HIV status and WAIS score (a–b) on subcortical thickness. Fig. 4(c–d) indicates that WAIS is significantly positively associated with left caudate thickness in the control group. Fig. 4(a–b) indicates that the magnitude of the association is significantly reduced in the HIV + cohort. The direction of the association between thickness and WAIS score is shown to be inverse in (e–f), which maps the association only within HIV + participants. No significant associations between shape and WRAT score were observed.

### 3.8 HIV status classification

**Fig. 5:**
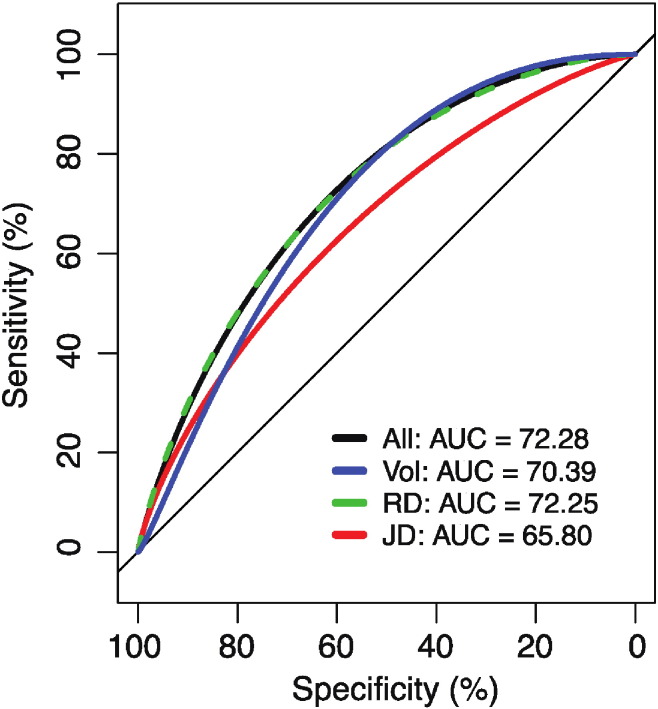
ROC curves and associated AUC values for random forest classification using the full set of morphological features. ROCs for feature subsets are also provided.

Classification of HIV status using the full set of morphology descriptors with a 2-fold cross validation yielded an area under the receiver operating characteristic (ROC) curve (AUC) of 72.3% (q = 0. 025). We also used a more computationally expensive leave-one-out cross validation, but this did not improve our classifier's performance, giving an AUC of 71.9%. Again, using 2-fold cross validation, we investigated the performance of feature subsets comprised exclusively of one of, volume, RD or JD features. Fig. 5 plots the ROC curves for each set of classifier inputs. The volumetric subset afforded an AUC of 70.4% (q = 0.025); RD 72.3% (q = 0. 025) and JD 65.8% (q = 0.080).

**Fig. 6:**
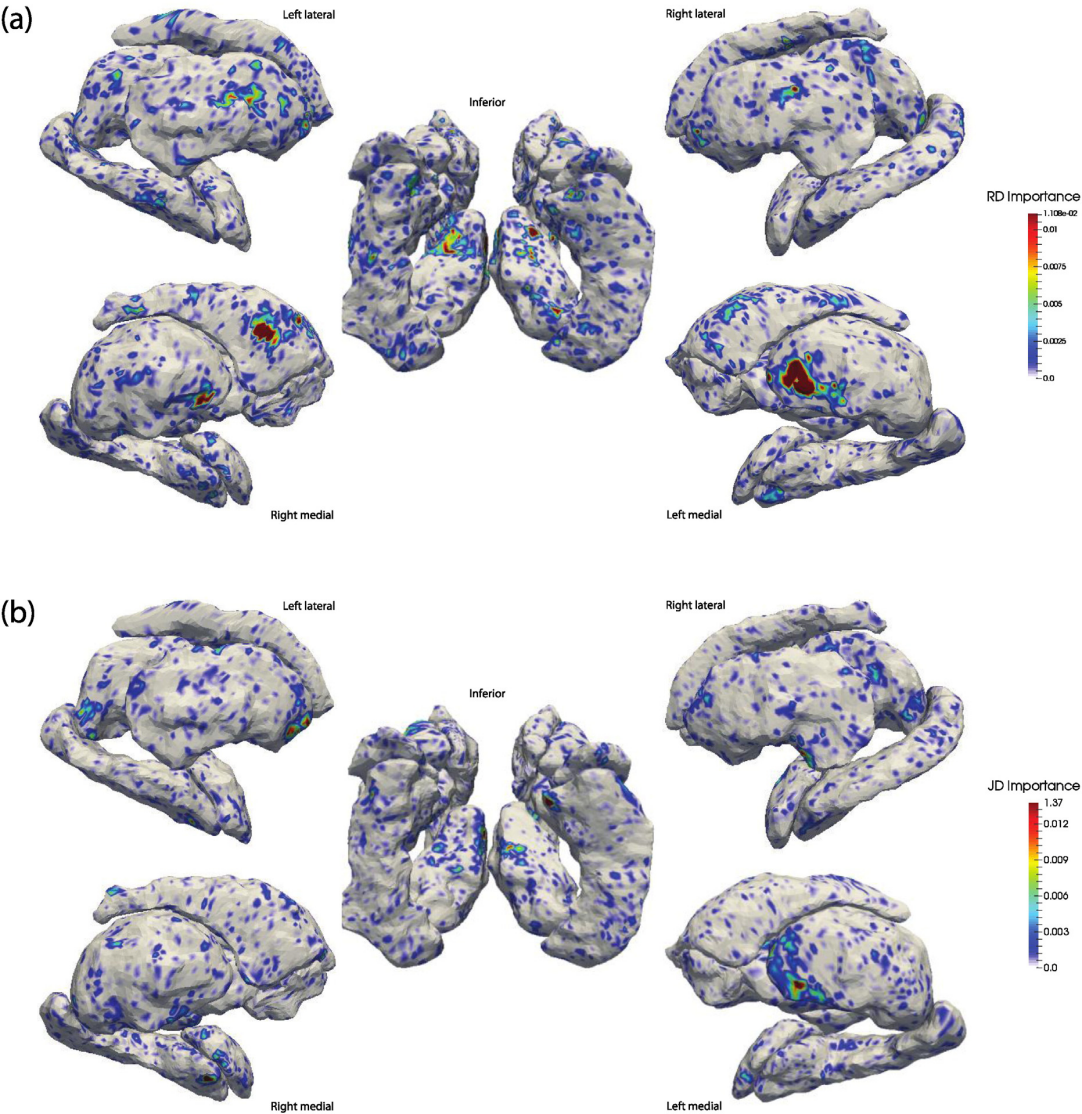
Mapping of random forest importance scores in the classification of HIV status to subcortical surfaces. As all features were included in the same RF model, their importance is with respect to all other features. (a) RD and (b) JD feature set importance scores. Images are in radiological orientation (i.e. left–right flipped), orientations are provided beside each set of surfaces.

**Fig. 7:**
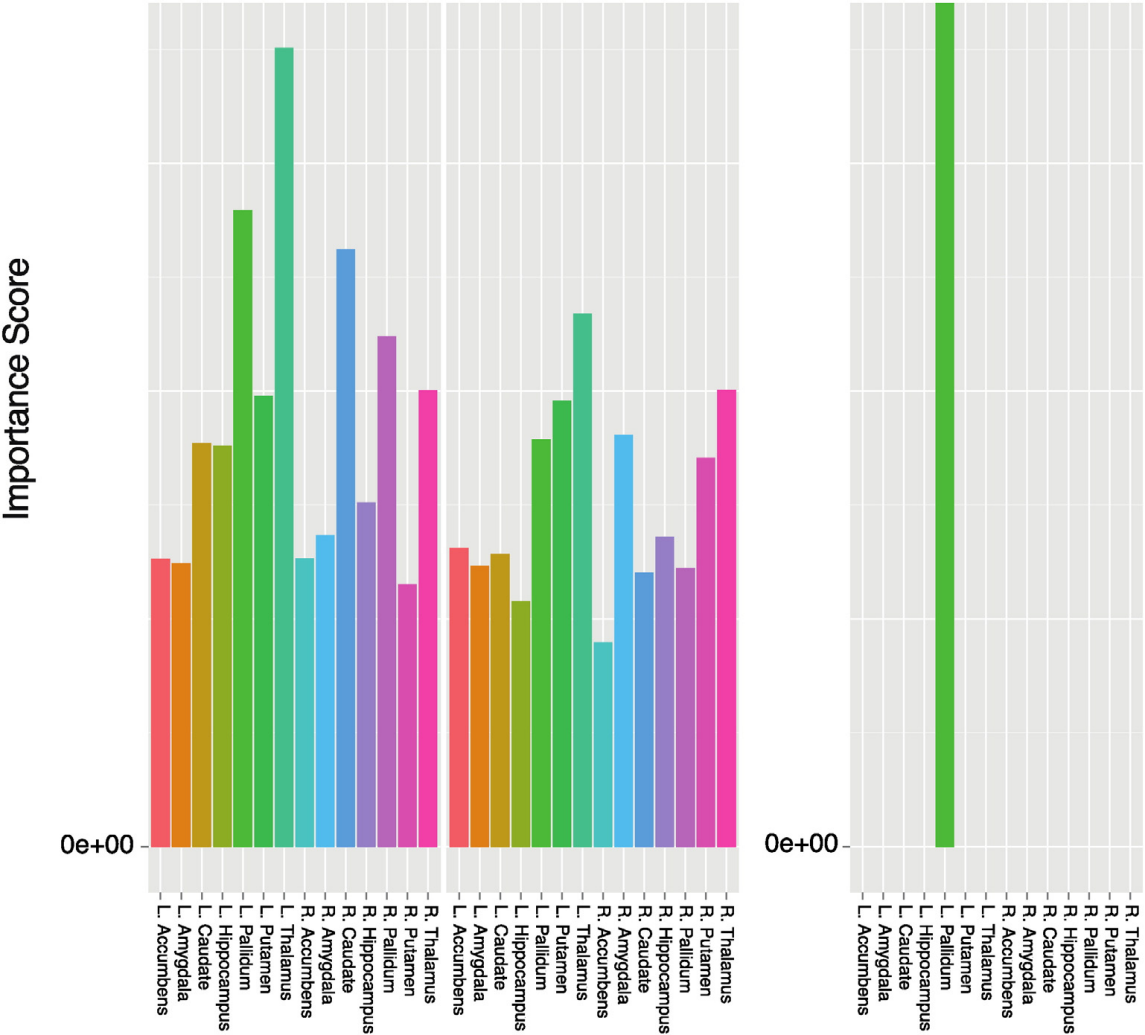
Average random forest importance scores by region and feature set.

In Fig. 6 we mapped the importance scores of each RD and JD value derived from the RF model to the corresponding surface location, providing a visual representation of how each region's shape-based morphometry drove the RF model. Fig. 7 complements this by plotting the importance score for each region; RD and JD-based importance scores were averaged within each surface. Fig. 7 indicates that RD features were weighted more heavily than their JD counterparts. We also note that the only volumetric measure that was relevant to the RF was the left pallidum. Comparing Fig. 6, Fig. 2, there is widespread correspondence between regions considered important to the RF classifier and regions that were statistically significant in modeling the effects of HIV. While this is perhaps not surprising, it serves to validate both sets of findings.

## 4 Discussion

The development of cART has allowed for HIV infected individuals to live to advanced ages and as a result it is estimated that the proportion of HIV + people over the age of 45 years is close to 50% (Collaboration, A.T.C., 2008) and the demographic of over 55 years is the most rapidly growing among those with HIV (Cohen et al., 2015, Hall et al., 2008). While cardiovascular diseases such as stroke and hypertension (Gorelick et al., 2011) as well as depression (Vink et al., 2009) and diabetes (McBean et al., 2004) are all common comorbidities associated with aging and may contribute to neurodegeneration, accumulating evidence indicates that HIV exacerbates and uniquely contributes to age-related neurodegeneration (Holt et al., 2012). Several lines of evidence have also suggested that particular cART therapies may have neurotoxic properties that affect cognition and brain aging (Ances et al., 2008, Robertson et al., 2010, Schweinsburg et al., 2005). The advancing age of the HIV + population and the suggested viral contribution to the aging process underlies the importance of characterizing the progression of brain atrophy in the older HIV population. The development of biomarkers to track the extent of the viral effects in the central nervous system may help to further inform clinicians in selecting the appropriate line of therapy for a particular patient. Using standard volumetric and novel surface-based shape descriptors we were able to identify several important HIV-related patterns of brain abnormalities.

Previous studies have noted that HIV has a proclivity for subcortical regions such as the basal ganglia (Aylward et al., 1993, Aylward et al., 1995, Berger and Nath, 2000, Gottumukkala et al., 2014) where the virus may replicate and maintain a reservoir despite active cART therapy. Atrophy of these subcortical regions is commonly reported in HIV-infected individuals. Other reported abnormalities of subcortical structures include hypermetabolism of the thalamus and basal ganglia (Drevets, 2001), a reduction in dopaminergic transporters in the putamen and striatum and increased mean diffusion in the putamen (Chang et al., 2008). Corroborating these reported effects, we observed that HIV positivity was associated with greater subcortical atrophy, particularly in the caudate, putamen, pallidum, hippocampus and callosum.

Unexpectedly, we observed a dilation in the anterior aspect of the right pallidum in relation to extended TSD. Several subjects in our HIV + cohort had a history of methamphetamine use which has been linked to enlarged subcortical volumes (Chang et al., 2005, Jernigan et al., 2005), however we did not observe an association between drug abuse history and subcortical morphometry. Another possible concern is that TSD could be confounded by participant age. However, this seems unlikely given the relatively low standard deviation of age among the HIV + cohort (SD = 4.57).

WAIS scores were differentially associated with the thickness of the left caudate with a positive association within the control group and a negative association within the HIV + group. The reason for this inverse association remains unclear.

We expected to see patterns of morphometry associated with common clinical measures linked to HIV such as viral load and nadir CD4 count, but none of these associations were significant. This may be due, in part, to limited power, as all subjects were on cART with relatively high CD4 counts and either undetectable or very low plasma HIV RNA levels. To alleviate this, our ENIGMA consortium is beginning a larger-scale project to relate clinical markers of HIV disease burden to brain measures across multiple cohorts (Jean-Paul Fouche et al., 2015).

In addition to mapping abnormal subcortical morphometry, we used the morphological descriptors to predict HIV status in a RF framework. While a simpler logistic classifier was considered the dimensionality of our feature set renders these simpler models less tractable and required initial feature selection and feature scaling to allow convergence. Given the requirement of extra steps for a logistic model and the natural added value of importance scores, the RF was a natural choice. This type of predictive framework helps to verify that observed group differences robustly distinguish HIV status. Given the precision of a blood test to diagnose HIV, classifiers based on brain morphometry are not meant to identify infected individuals; rather the added benefit is the identification of potential biomarkers of interest to track the progression of the deleterious viral effects in the brain. In mapping the importance of each surface-based feature derived from the RF back onto the subcortical surfaces, we were able to observe a large degree of correspondence between thresholded statistical maps and feature importance. This correspondence between importance weightings and statistically differing regions mutually validates the observed profile of changes in HIV participants as well as the potential to use these regions as biomarkers for HIV's effect in subcortical brain regions. The exclusive use of RD features moderately outperformed the exclusive use of volume as predictors, both of which outperformed the JD-only classifier though no feature-specific classifiers outperformed another at the alpha = 0.05 level of significance.

Analysis of the importance scores resulting from the random forest suggests several things. First, we observed that RD (thickness) features were on average more important than JD or volumetric features. In particular thickness of the left thalamus, left pallidum and right caudate were highly important in discerning HIV status. Patterns of importance were similar for JD features but to lesser extents than RD. The volume of the left pallidum was the single most informative feature; yet no other volumetric measures were considered important after taking into account the shape descriptors.

Because the present study is not longitudinal in design, we are unable to address questions of accelerated brain aging caused by HIV. However, we do observe widespread yet concentrated regions of atrophy in the subcortical regions of interest among HIV + participants that are age disproportionate. The primary regions of advanced atrophy seem concentrated in the basal ganglia, thalamus and caudate. This is a departure from atrophy patterns observed in common forms of neurodegeneration such as Alzheimer's disease which principally targets hippocampal regions. However, as previously noted, increased atrophy of subcortical brain regions has been widely reported in HIV neuroimaging literature. Several studies have also observed decreased cognitive functions in vertically infected children that increase with disease severity (Chase et al., 2000, Le Doare et al., 2012, Smith et al., 2006) suggesting that neurological dysfunction is a factor in infection at any age. It would be tenuous to directly compare observed differences between these age groups since study designs and protocols differ widely.

We were notably unable to identify a relationship between brain morphometry and viral load, nCD4 count, HAND status or drug abuse history. As this is a cohort of elderly subjects, many of whom were infected prior to the development of modern cART yet managed to survive, the survivorship effect may generally limit our abilities to find such associations; that is it is possible that these individuals are generally healthier. This also limits our ability to generalize these findings to a younger HIV + population. nCD4 counts have previously been linked to lower regional brain volumes (Hua et al., 2013) in a younger cohort. Our lack of observed associations with HAND status also likely reflects the fact that none of our cohort had developed HAD; rather 14 had ANI while 16 had MND, far milder forms of HAND. Drug abuse history was similarly limited by the fact that few of our subjects had a history of drug abuse and those that did were distributed among different classes of drugs.

## 5 Conclusions

We have characterized patterns of abnormal brain morphometry in an elderly HIV + cohort. The increased atrophy observed in HIV + patients lends support to the idea that HIV contributes to age-disproportionate brain aging. Regions of atrophy were localized by vertex wise descriptions of thickness, dilation and contraction. Pronounced abnormalities were found in the medial head of the caudate, the bilateral pallidum and the putamen and the thalamus. Several other regions exhibited significant abnormalities as well. Ventricular spaces were enlarged in the HIV + cohort. We also observed a paradoxically positive association between the TSD and the size of the right anterior pallidum that was not attributable to substance abuse history. This effect may be a product of medication use or even the survivorship effect, however neither effect can be evaluated with this cohort as all are on stable cART and, of course, survivors. Infection status was classifiable using only subcortical morphometry with an AUC of 72.3%. Classification was driven heavily by the thickness of pallidal, thalamic and caudate surfaces. Future studies should further develop the use of these descriptors to aid in tracking the progression of HIV's effect on the aging brain.

## Conflict of interest

The other authors have no disclosures related to the study.

## Figures and Tables

**Table 1:** Demographic and clinical characteristics.

	Patients, N = 63	Controls, N = 31
*Demographics*		
Age, mean (SD), y	64.68 (4.57)	65.35 (2.21)
Gender (M/F)	61/2	27/4
Adjusted education, years*	16.09 (2.25)	17.53 (2.09)

*Clinical information*		
CD4, mean (SD), cells/mm^3^	516.44 (212.30)	–
Nadir CD4, mean (SD), cells/mm^3^	204.96 (154.85)	–
Time since diagnosis, mean (SD), y	20.39 (6.31)	–
Viral load, mean (SD), copies/mm^3^	16,380.58 (76,418.68)	–
Detectable viral load, yes/no	24/39	–
Receiving cART, yes/no	63/0	–
History of diabetes	8	0
History of depression*	28	8
APOE allele, yes/no/unknown	12/51/0	6/13/12

*Cognitive information*		
ANI	14	–
MND	16	–
HAD	0	–
WAIS score, mean (SD)*	48.77 (10.73)	57.27 (7.48)
WRAT score, mean (SD)*	64.05 (4.26)	66.50 (1.93)
Substance abuse history	none/recent/remote	
Marijuana	61/0/2	–
Cocaine	55/0/8	–
Crack	62/0/1	–
Meth	57/0/6	–
Alcohol	50/0/13	–

Abbreviations: ANI = Asymptomatic Neurocognitive Impairment; MND = Mild Neurocognitive Disorder; HIV-associated Dementia. *Significant difference between HIV status. WAIS = Wechsler Adult Intelligence Scale; scores available for N = 57 patients and N = 22 controls. WRAT = Wide Range Achievement Test; scores available for N = 57 patients and N = 20 controls.
